# Supplementary material for: perfectphyloR: An R package for reconstructing perfect phylogenies
Source: BMC Bioinformatics. 2019 Dec 23;20:729. doi: 10.1186/s12859-019-3313-4 (PMC6929499; doi:10.1186/s12859-019-3313-4)
Supplement: Supplementary file 1 — Additional file 1 testDendAssoRI(): Arguments and example call [file 12859_2019_3313_MOESM1_ESM.pdf]

`testDendAssoRI()`: Arguments and example call

The function `testDendAssoRI()` has five key arguments:

1. `rdend`: An `ape` multiphylo object of reconstructed dendrograms at each focal SNV.
2. `cdend`: An `ape` phylo object of the comparator dendrogram.
3. `hapMat`: An object of class `hapMat` containing SNV sequences.
4. `k`: An integer that specifies the number of clusters that the dendrogram should be cut into. The default is `k = 2`. Clusters are defined by starting from the root of the dendrogram, moving towards the tips and cutting across when the appropriate number of clusters is reached.
5. `nperm`: Number of permutations for the test of any association across the genomic region. The default is `nperm = 0`; i.e., association will not be tested.

To illustrate, we use the example dataset `ex_hapMat_data` with 200 sequences and 2747 SNVs. We plot the Rand index values summarizing the association between the comparator dendrogram at SNV position 975 kilobase pairs and the reconstructed dendrogram at each SNV position across the 2 Mbp genomic region (Figure 2a).

```
R> # Comparator true dendrogram at 975 kbp.
   data(tdend)

   # hapMat data object.
   data(ex_hapMat_data)

   # Reconstruct dendrograms across the region.
   allrdends <- reconstructPPregion(hapMat = ex_hapMat_data,
                                 minWindow = 55)

   # Rand index profile based on 6 clusters.
   RI_profile <- testDendAssoRI(rdend = allrdends,
                                cdend = tdend,
                                    k = 6,
                               hapMat = ex_hapMat_data,
                                nperm = 1000,
                                 xlab = "SNV positions (bp)",
                                 ylab = "Rand indices",
                                 main = "Association Profile")

    # Omnibus P value for overall associaion.
    RI_profile$OmPval
    [1] 0.000999001
```
